# The F_1_F_o_-ATP Synthase β Subunit Is Required for *Candida albicans* Pathogenicity Due to Its Role in Carbon Flexibility

**DOI:** 10.3389/fmicb.2018.01025

**Published:** 2018-05-23

**Authors:** Shui-Xiu Li, Hao-Tian Wu, Yu-Ting Liu, Yi-Ying Jiang, Yi-Shan Zhang, Wei-Da Liu, Kun-Ju Zhu, Dong-Mei Li, Hong Zhang

**Affiliations:** ^1^Department of Dermatology, The First Affiliated Hospital of Jinan University, Guangzhou, China; ^2^Institute of Mycology, Jinan University, Guangzhou, China; ^3^Jiangsu Key Laboratory of Molecular Biology for Skin Diseases and STIs, Institute of Dermatology, Chinese Academy of Medical Sciences, Nanjing, China; ^4^Department of Microbiology and Immunology, Georgetown University Medical Center, Washington, DC, United States

**Keywords:** F_1_F_o_-ATP synthase, β subunit, *Candida albicans*, pathogenicity, carbon flexibility

## Abstract

Previous work has explored link between mitochondrial biology and fungal pathogenicity in F_1_F_o_-ATP synthase in *Candida albicans*. In this work we have detailed the more specific roles of the F_1_F_o_-ATP synthase β subunit, a key protein subunit of F_1_F_o_-ATP synthase. The ability to assimilate alternative carbons in glucose-limited host niches is known to be a critical factor for infection caused by opportunistic pathogens including *C. albicans*. The function of the F_1_F_o_-ATP synthase β subunit was characterized through the construction of an *ATP2* gene null mutant (*atp2*Δ/Δ) and the gene-reconstituted strain (*atp2*Δ/*ATP2*) in order to understand the link between carbon metabolism and *C. albicans* pathogenesis. Cell growth, viability, cellular ATP content, mitochondrial membrane potential (ΔΨm), and intracellular ROS were compared between null mutant and control strain. Results showed that growth of the *atp2*Δ/Δ mutant in synthetic medium was slower than in complex medium. However, the synthetic medium delayed the onset of reduced cell viability and kept cellular ATP content from becoming fully depleted. Consistent with these observations, we identified transcriptional changes in metabolic response that activated other ATP-generating pathways, thereby improving cell viability during the initial phase. Unlike glucose effects, the *atp2*Δ/Δ mutant exhibited an immediate and sharp reduction in cell viability on non-fermentable carbon sources, consistent with an immediate depletion of cellular ATP content. Along with a reduced viability in non-fermentable carbon sources, the *atp2*Δ/Δ mutant displayed avirulence in a murine model of disseminated candidiasis as well as lower fungal loads in mouse organs. Regardless of the medium, however, a decrease in mitochondrial membrane potential (ΔΨm) was found in the *atp2*Δ/Δ mutant but ROS levels remained in the normal range. These results suggest that the F_1_F_o_-ATP synthase β subunit is required for *C. albicans* pathogenicity and operates by affecting metabolic flexibility in carbon consumption.

## Introduction

*Candida albicans* is the most common cause of disseminated candidiasis (Pfaller et al., 2014). The mortality rate remains high (∼40%) even though antifungal azoles have been used for several decades (Brown et al., 2012; Pfaller et al., 2014).

Mitochondria play an important role in *C. albicans* pathogenicity (Shingu-Vazquez and Traven, 2011; Qu et al., 2012; Calderone et al., 2015). The F_1_F_o_-ATP synthase is the Complex V (CV) of mitochondrial electron transport chain (ETC). This protein complex is composed of α_3_β_3_γδ𝜀ab_2_c10–15 subunits and is a key enzyme of cell bioenergetics. The primary role of CV is to convert the electrochemical gradient (created by other ETC complexes across the mitochondrial inner membrane) into ATP that is used for all living organisms. The β subunit (*ATP2*) is a part of the catalytic core F_1_F_o_-ATP synthase.

The ability to assimilate alternative carbons in glucose-limited host niches has been known as a critical factor for infection caused by opportunistic pathogens including *C. albicans* (Lorenz and Fink, 2001; Vylkova et al., 2011; Lorenz, 2013; Cowen et al., 2017), even though these alternative carbon sources may not been preferred for energy production. Unlike the model yeast *Saccharomyces cerevisiae*, *C. albicans* has two or three respiration routes and fermentative pathways that make this pathogen a potent competitor in the host niches. However, the survival mechanisms that *C. albicans* employs to cope with glucose and alternative carbon assimilation are poorly understood.

Understanding the role of the β subunit of F_1_F_o_-ATP synthase has an important implication in explaining carbon metabolism during *C. albicans* infection. The study on *Mycobacterium tuberculosis* has shown that growing on non-fermentable carbon sources is more rapidly killed by inhibiting F_1_F_o_-ATP synthase than when growing on fermentable carbon sources (Koul et al., 2014). Some bacteria have even evolved an alternative metabolic mechanism whenever an absence or inhibition of classical respiration occurs during their growth on fermentable carbon sources such as glucose, e.g., substrate-level phosphorylation (Jensen and Michelsen, 1992; Santana et al., 1994; Cook et al., 2014). In *S. cerevisiae*, a deletion mutation in the CV causes a defect in oxidative phosphorylation that increases the difficulty for this organism to grow on media with non-fermentable carbon sources (Lai-Zhang et al., 1999). By contrast, little is known about the metabolic responses of *C. albicans* with regard to any lack of or inhibition of this protein complex, especially in different carbon sources.

In this study, we aim to understand the pathogenic roles of the β subunit of F_1_F_o_-ATP synthase in *C. albicans* and its likely mechanisms. With a genetic null mutant (*atp2*Δ/Δ) and a gene-reconstituted strain (*atp2*Δ*/ATP2*), we discover that the *atp2*Δ/Δ mutant tends to enhance the activities of other ATP-generating pathways in order to maintain cell viability under fermentable carbon source conditions. However, failure to utilize non-fermentable carbon sources in the *atp2*Δ/Δ mutant results in immediately and sharply reduced cell viability. At the same time *in vivo* studies demonstrate that the *atp2*Δ/Δ mutant displays avirulence in disseminated mice model. We conclude that one function of the β subunit of F_1_F_o_-ATP synthase is to assist carbon flexibility for keeping *C. albicans* pathogenicity.

## Materials and Methods

### Strains and Growth Conditions

For routine propagation, strains were grown in YPD broth or on YPD agar (1% yeast extract, 2% peptone, and 2% glucose) with or without compounds as indicated. Selection for nourseothricin-resistant strains, YPD with 200 μg/mL of nourseothricin (YPD-Nou) was used (Reuss et al., 2004).

*Candida albicans* SC5314 (wild type) (Gillum et al., 1984) was used to generate the *atp2*Δ/Δ mutant (*atp2*Δ*::FRT*/*atp2*Δ*::FRT*) and *atp2*Δ*/ATP2* gene-reconstituted strain (*atp2*Δ*::FRT* /*ATP2*::FRT) (Supplementary Table S1). A null mutant of *ATP2* (*atp2*Δ/Δ) was constructed by using the *SAT1* flipper methodology (Reuss et al., 2004; Sasse and Morschhauser, 2012). Briefly, approximately 400 bp of homology immediately 5′ or 3′ of the *ATP2* ORF were amplified by PCR and cloned between the ApaI/XhoI and SacI/SacII sites, respectively, of pSFS2. The construction of deletion cassette was based on plasmid pSFS2, which was genetically cloned approximately 400 bp up- and down-stream of *ATP2* ORF. The flanking up- and down-stream ranges of *ATP2* were amplified by PCR and introduced into ApaI/XhoI and SacI/SacII sites of pSFS2. The resulting *SAT1*-FLP cassette was linearized with SacI and used to transform *C. albicans* SC5314, with selection on YPD-Nou. Genomic DNA was isolated, and cassette integration confirmed in the selected candidates via PCR. Nourseothricin sensitivity was restored by inducing the expression of the Mal2p-FLP recombinase gene through growth on yeast extract-peptone-maltose medium. This process was repeated to generate the second allele disruption of *atp2*Δ/Δ (*atp2*Δ*::FRT*/*atp2*Δ*::FRT*). For reconstituted strain, one allele complementation of the mutant strain was done with plasmid pSFS2-down that carries the downstream approximately 400 bp of *ATP2* ORF. The entire *ATP2* ORF with flanking upstream and downstream approximately 400 bp were amplified by PCR and cloned between the ApaI and XhoI of pSFS2-down. This plasmid was linearized with SacI and used to transform *atp2*Δ/Δ mutant strain to generate the *atp2*Δ*/ATP2* gene-reconstituted strain (*atp2*Δ*::FRT* /*ATP2*::*FRT*).

The primers used for gene deletion in this study are listed in Supplementary Table S2. The confirmation of the *atp2*Δ/Δ mutant and reconstituted strain (*atp2*Δ/*ATP2)* are performed via nested PCR and Southern blot as shown in Supplementary Figures S1–S6.

### Growth Curve and Generation Time Assay

All strains were routinely grown in 5 mL of YPD at 30°C overnight, washed with PBS, then inoculated in 100 mL of rich (YPD) or minimal (YNB) media with an OD_600_ of 0.02. Shake cultures were grown at 30°C and OD_600_ of each strain was measured every hour. And the generation times were calculated (Bambach et al., 2009). All experiments were performed in triplicate on two separate days. *P*-values were determined using the unpaired Student’s *t*-test.

### Cell Viability Assay

To determine if cell viability in stationary phase is affected in the *atp2*Δ/Δ, all strains were growth in both rich (YPD) or minimal (YNB) media with, respectively, 2% glucose, or YNB medium with 2% glucose, 2% glycerol, 2% ethanol, 2% oleic acid, 2% citrate, and 2% acetate. Cells of each strain were collected daily for 30 days, to determine the OD_600_, and plated on YPD agar for 48 h to enumerate the cfu/mL (Koul et al., 2014; She et al., 2015). All experiments were performed in triplicate on two separate days.

### Spot Assay

To investigate the growth of *C. albicans* strains to different carbons source, cells were overnight cultured, washed and suspended in PBS with an initial OD_600_ of 1.0. A aliquot of 5 μL of 10-fold dilutions was spotted onto YP agar with 2% glucose, 2% glycerol, 2% ethanol, 2% oleic acid, 2% citrate, and 2% acetate. Plates were cultured at 30°C for 2 days and then photographed (Li et al., 2017). All experiments were performed in triplicate on two separate days.

### Illumina Sequencing and Analysis of RNAseq Data

The contribution of F_1_F_o_-ATP synthase gene *ATP2* to transcriptional events was determined. RNA isolation and Illumina sequencing were performed as described previously (Guo et al., 2014; She et al., 2015). All strains were grown in 50 mL of 2% SD medium at 30°C for either 5 h for WT and reconstituted strains or 9 h for *atp2*Δ/Δ mutant. Three biological replicates were used to extract RNA from each strain. Then the cleaved RNA fragments were constructed into the final cDNA library in accordance with the protocol for the Illumina RNA ligation based method (Illumina, San Diego, CA, United States). And then we performed the single end sequencing on an Illumina Hiseq2000/2500 at the (LC Sciences, United States) following the vendor’s recommended protocol.

Reads were then aligned to the reference genome and gene model annotation files of *C. albicans* (GenBank: gca_000784635) using the software program Bowtie 2 (v2.1.0) (Langmead and Salzberg, 2012). The number of clean reads corresponding to each gene was calculated and normalized to the number of Reads Per Kilobase of exon model per million mapped reads (RPKM) (Mortazavi et al., 2008). Based on the expression levels, differential expression genes (DEGs) analysis (*atp2*Δ/Δ vs. WT*, atp2*Δ*/ATP2* vs. WT) using DESeq2 (Love et al., 2014). The corrected *P*-value (*Q*-value), False Discovery Rate (FDR), was used to screen the DEGs; “*P* ≤ 0.05 and absolute value of log_2_ (fold change) ≥ 1” was set as the threshold to judge significance of differential gene expression.

### Mitochondrial Membrane Potential Measurement

The cyanine dye JC-1 was used for determination of mitochondrial membrane potential of each strain (Li et al., 2017). All strains were grown in YPD or YNB overnight at 30°C, washed and suspended with PBS. A suspension of 2.0 × 10^6^ cells was incubated with 5 μM JC-1 at 37°C for 15 min in the dark. Cells were washed three times, resuspended in PBS buffer, and measured in FACScan flow cytometer. The excitation spectra at 595 nm was used in FACScan flow cytometer with emission spectra at 488 nm (Becton Dickinson) to determine the mitochondrial membrane potential. All experiments were performed in triplicate on two separate days. *P*-values were determined using the unpaired Student’s *t*-test.

### Cellular ATP Content Assay

The cellular ATP content was determined as described previously (Guo et al., 2014). All strains are growth in YNB media with 2% glucose or 2% glycerol, 2% ethanol, 2% oleic acid, 2% citrate, and 2% acetate. An aliquot of 2 × 10^7^ cells from each strain was mixed with the same volume of BacTiter-Glo^TM^ reagent (Promega Corporation, Madison, WI, United States) and incubated for 15 min at room temperature in the dark. Luminescent signals were determined on a full wavelength multifunctional enzyme mark instrument (Thermo Scientific). A control tube without cells was used to obtain a value for background luminescence. A standard curve of incremental ATP concentrations (from 1 mM to 10 pM) was constructed. Signals represented the mean of three separate experiments and the ATP content was calculated from the standard curve. All experiments were performed in triplicate on two separate days.

### Endogenous ROS Measurement

For determination of endogenous ROS levels, all strains were grown in YPD or YNB overnight at 30°C, washed and suspended with PBS, 1 × 10^7^ cells of each strain were stained with DCFDA (20 μg/mL) at 37°C for 20 min in the dark for measuring intracellular ROS. The emission spectra at 488 nm and excitation spectra at 595 nm were determined by FACScan flow cytometer (Becton Dickinson). ROS levels were determined from triplicate samples of each strain (She et al., 2015). *P*-values were determined using the unpaired Student’s *t*-test.

### NADH/NAD^+^ Ratio Assay

For determination of NADH/NAD^+^ ratio, all strains were grown in YPD at 30°C. Logarithmically growing cells were washed three times with pre-warmed sterile PBS, resuspended in the same volume of YNB supplemented with 2% glucose, and continued to incubate at 30°C for 24 h. The NAD^+^ and NADH contents were measured using NAD^+^/NADH-Glo^TM^ Assay (Promega Corporation, Madison, WI, United States). Luminescent signals were determined on a full wavelength multifunctional enzyme mark instrument (Thermo Scientific). NADH/NAD^+^ ratio was calculated. All experiments were performed in triplicate on two separate days. *P*-values were determined using the unpaired Student’s *t*-test.

### Effect of *ATP2* on the Virulence in Mice

A mouse model of disseminated candidiasis was used to evaluate the virulence of the strains (Spellberg et al., 2003, 2005). Female BALB/c mice (18–22 g; Guangdong Medical Laboratory Animal Center, Foshan, Guangdong, China) were used for all experiments. Mice were injected via the lateral tail vein with either a suspension of 1 × 10^6^ cells from each strain. Survival rate was calculated from 10 infected mice per strain. For determination of fungal burden, another three mice from each group were euthanized after 1, 24, 48, and 72 h infection. Kidney, spleen, and liver were harvested, weighed, homogenized, and quantitatively cultured. In addition, at day 1 of infection, mice were killed and organs removed to fix in 10% buffered formalin, then embed in paraffin, sectioned and stained with Periodic Acid-Schiff for histological study. Mortality was represented with Kaplan-Meier survival curves and quantitative tissue burdens were marked in the log scale and compared in the Mann–Whitney test.

The animal experiments were performed under the guidance of a protocol approved by the Animal Study Committee of the Institute of Dermatology, CAMS, according to the National Guidelines for Animal Care. All animal experiments were carried out with permission from the Ethical Committee of Institute of Zoonosis, Jinan University, Guangzhou, China (Ref No. 20080101).

## Results

### The Generation Time Is Increased in the *atp2*Δ/Δ Mutant

**FIGURE 1:**
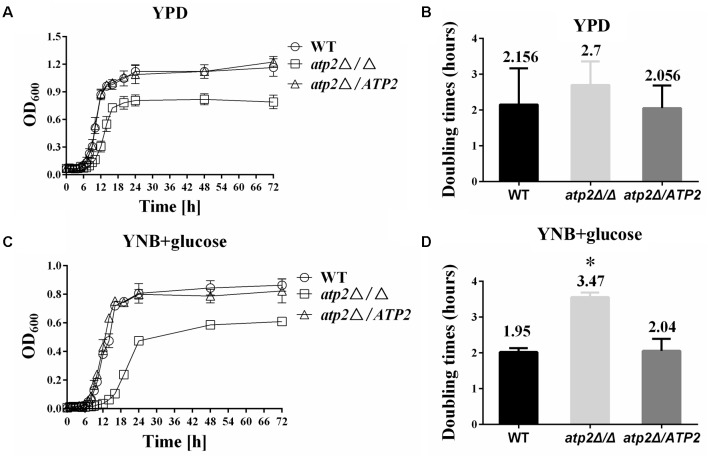
Deletion of *ATP2* results in a longer generation time. The growth curves of strains WT, *atp2*Δ/Δ and *atp2*Δ/*ATP2* are shown. Strains were grown overnight in YPD, transferred to fresh rich (YPD) or minimal (YNB) media at an OD_600_ of 0.02, and incubated at 30°C. The absorbance was measured every 1 h for 48 h and the generation times were calculated. The growth **(A,C)** (OD_600_) and the generation times **(B,D)** of all strains were monitored when cultures were grown on rich **(A,B)** and minimal media **(C,D)**. The growth curves represent the averages of three experiments and three replicates per time point per strain. ^∗^*P <* 0.05 compared with WT. Representative results from at least three experiments are shown.

The *atp2*Δ/Δ mutant displayed moderately reduced growth rates *in vitro* when grown both in rich medium (YPD) and minimal medium (YNB), demonstrated by a longer lag and a lower maximum growth at stationary phase than WT or gene-reconstituted (*atp2*Δ*/ATP2*) strains during a 48 h incubation course (**Figures 1A,C**). In YPD medium, the doubling time of the *atp2*Δ/Δ mutant (2.7 h) is not significantly different from the doubling times of WT (2.156 h) (*p* > 0.05) and the *atp2*Δ*/ATP2* strains (2.056 h) (*p* > 0.05), respectively, as shown in **Figure 1B**. In minimal medium, when the WT and *atp2*Δ*/ATP2* strains doubled at 1.95 and 2.04 h, respectively (*p* > 0.05), the *atp2*Δ/Δ mutant grew much more slowly, with a doubling time of 3.47 h, which is significant (*p* < 0.05) when compared with WT and *atp2*Δ*/ATP2* strains (**Figure 1D**). Nevertheless, the *atp2*Δ/Δ mutant showed a distinctly lower maximum growth in both media. These results suggest that *ATP2* is important for optimum growth in both nutrients environment, especially in minimal medium.

### The Cell Viability Is Reduced in the *atp2*Δ/Δ Mutant

**FIGURE 2:**
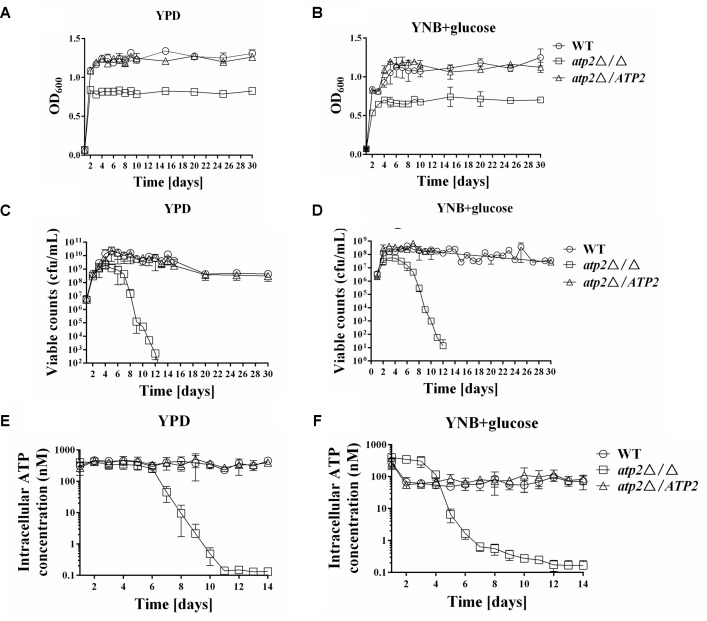
The cell viability is reduced in the *atp2*Δ/Δ. The growth **(A,B)** (OD_600_), viability **(C,D)** (cfu/mL) and cellular ATP content **(E,F)** (nM) of all strains were monitored when cultures were grown on rich (YPD) media **(A,C,E)** or minimal (YNB) media **(B,D,F)**. Representative results from at least three experiments are shown.

To determine whether the decreased density of cell growth in the *atp2*Δ/Δ at stationary phase was caused by lower cell viability, the cell survival rates were determined in all strains. We found that the maximum growth at stationary phase and cell viability of the *atp2*Δ/Δ was significantly decreased in both YPD and YNB media with 2% glucose (**Figures 2A–D**), particularly after 5 days in YPD and 5 days in YNB media. In the early stages, the *atp2*Δ/Δ had a comparable number of viable cells when compared with the control strain. However, *atp2*Δ/Δ viability began to decrease at day 5 in YPD medium and at day 5 in YNB medium, and then both exhibited a further sharp decline over the following 24 h by approximately 10-fold. By day 10, the cell viability of *atp2*Δ/Δ mutant was reduced by about 100,000-fold compared with WT in YPD. At day 13, no viable cells can be found in YPD but 10–100 cells can be seen in YNB. By contrast, the cell viability of WT or *atp2*Δ*/ATP2* lasted for at least 30 days without apparent changes. The sharply declined cell viability of the *atp2*Δ/Δ mutant in the late growth period indicates that *ATP2* is required to maintain cell viability in *C. albicans*, although minimal medium can delay the cell inviability.

### The Cellular ATP Content Is Reduced in the *atp2*Δ/Δ Mutant

The cellular ATP contents of each strain were initially measured in YPD and YNB media with 2% glucose. In agreement with the cell viability results, the cellular ATP content was reduced in the *atp2*Δ/Δ (**Figures 2E,F**). In YPD, the cellular ATP content in the *atp2*Δ/Δ was as the same as WT or *atp2*Δ*/ATP2* level during the first 5 days, but started to decline at day 6, and sharply declined at day 7 to an approximately fivefold compared with WT. Unexpectedly, the cellular ATP content in the *atp2*Δ/Δ grown in YNB was higher than those of WT and *atp2*Δ*/ATP2* during the first 3 days, as indicated by a 5.6-fold increase compared with WT. However, this high ATP production in the mutant did not last long. The cellular ATP content in the *atp2*Δ/Δ started to decline at day 4, and sharply declined at day 5 to an approximately 10-fold compared with WT. By day 10, the cellular ATP level of *atp2*Δ/Δ mutant was reduced 1550- and 130-fold in YPD and YNB, respectively. These data suggest that cellular ATP content may be part of the reason to interfere with *C. albicans* cell viability.

### Impact of Carbon Source on Cell Viability and ATP Production in the *atp2*Δ/Δ Mutant

**FIGURE 3:**
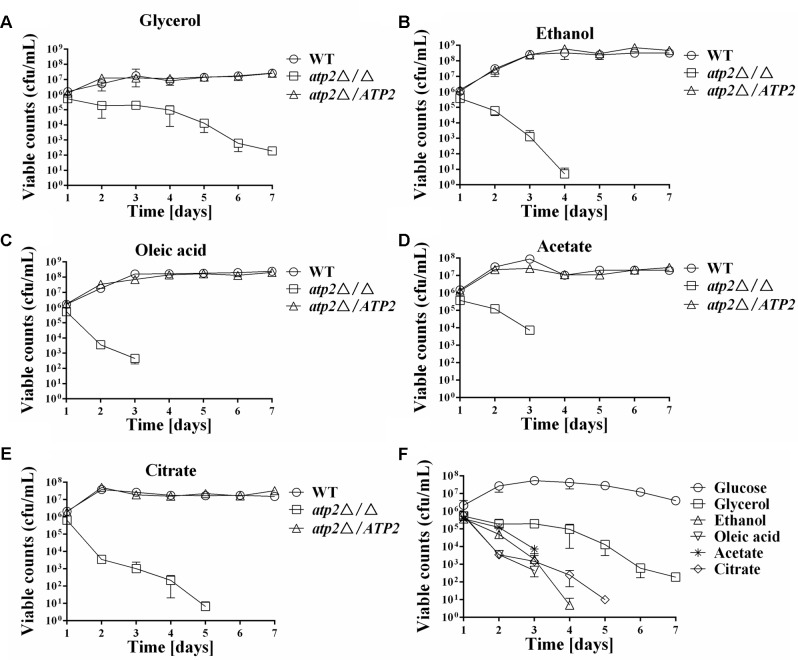
Impact of carbon source on cell viability of the *atp2*Δ/Δ. The cell viability (cfu/mL) of the *atp2*Δ/Δ mutant was compared to control strains grown on YNB medium with different non-fermentable carbon sources, such as glycerol **(A)**, ethanol **(B)**, oleic acid **(C)**, acetate **(D)**, and citrate **(E)**. The cell viability (cfu/mL) of the *atp2*Δ/Δ mutant in glucose was compared with the cell viabilities in non-fermentable carbon sources **(F)**. Representative results from at least three experiments are shown.

The effect of non-fermentable carbon source for cell viability was tested next. As depicted in **Figures 3A–E**, when grown on non-fermentable carbon sources such as glycerol, ethanol, oleic acid, citrate and acetate, the *atp2*Δ/Δ mutant displayed sharp declined cell viability as compared with WT and *atp2*Δ*/ATP2*; while cell viabilities of the *atp2*Δ*/ATP2* in these media were not different from WT. The different cell viability in WT and *atp2*Δ/Δ mutant suggests that oxidative phosphorylation is required for *C. albicans* survival in these non-fermentable carbon sources. When compared with survival pattern in glucose medium, cell viability of the *atp2*Δ/Δ mutant on each non-fermentable carbon source tended to be more sharply reduced (**Figure 3F**), particularly in the presence of sodium acetate. Among these non-fermentable carbon sources, killing enhancement was less pronounced than others. The carbon source dependent cell viability in the *atp2*Δ/Δ mutant suggests that glycolytic activity is extremely important for *C. albicans* survival.

**FIGURE 4:**
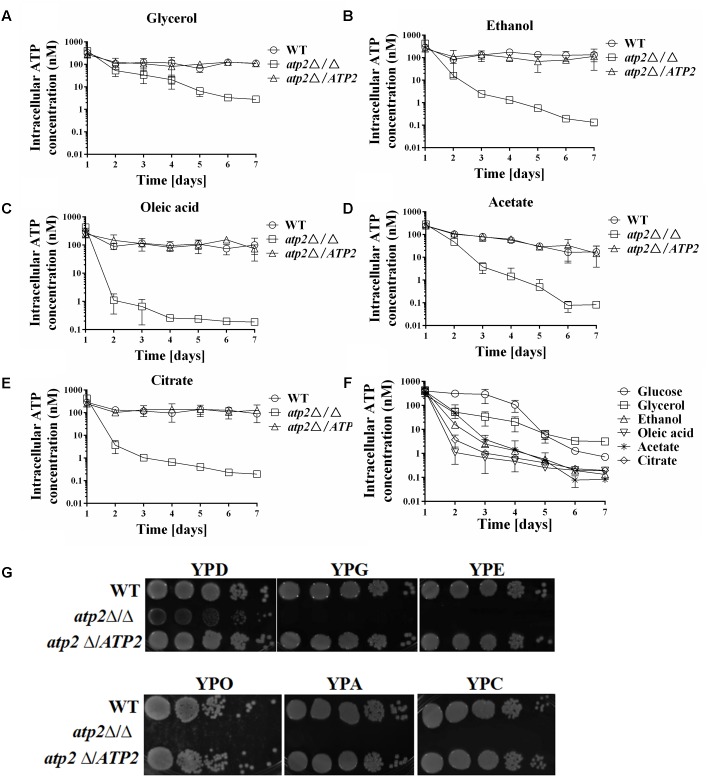
The cellular ATP content is gradually declined in the *atp2*Δ/Δ mutant on non-fermentable carbon sources. The cellular ATP content (nM) of all strains were monitored when cultures were grown on YNB medium with different non-fermentable carbon sources, such as glycerol **(A)**, ethanol **(B)**, oleic acid **(C)**, acetate **(D)** and citrate **(E)**. The cellular ATP content (nM) of the *atp2*Δ/Δ mutant in glucose medium was compared to ATP contents in variable non-fermentable carbon sources **(F)**. Representative results from at least three experiments are shown. Growth defects of the *atp2*Δ/Δ mutant on Non-fermentable carbon sources **(G)**. All strains were grown overnight in YPD medium, washed and serially diluted in PBS. Five microliters of each serial dilution was placed on the YP medium supplemented with 2% glucose (YPD), 2% glycerol (YPG), 2% ethanol (YPE), 2% oleic acid (YPO), 2% acetate (YPA), or 2% citrate (YPC) agar plates. All cultures were incubated for 2 days at 30°C. Representative results from at least three experiments are shown.

A WT level ATP is even harder to maintain for the *atp2*Δ/Δ mutant under non-fermentable carbon sources media. We observed that reduced ATP levels began earlier in the *atp2*Δ/Δ mutant in YNB medium with glycerol, ethanol, oleic acid, acetate, or citrate (**Figures 4A–E**) than in YNB medium with glucose. While the ATP levels remained high in first three days’ cultures of the *atp2*Δ/Δ mutant in YNB medium with glucose, the decreased ATP is clearly shown at 24 h, particular in YNB medium supplemented with oleic acid or citrate (**Figure 4F**). Such a sharp decline in the ATP profiling (>15-fold) explains the growth arrest of the *atp2*Δ/Δ mutant on the agar plates with each testing non-fermentable carbons in **Figure 4G** even though YP-based medium was used for analysis. On the other hand, it provides evidence that *ATP2* participates in carbon oxidation, especially non-glucose metabolic ATP production.

### The Mitochondrial Membrane Potential Decreases but the Intracellular ROS Is Not Altered in the *atp2*Δ/Δ Mutant

**FIGURE 5:**
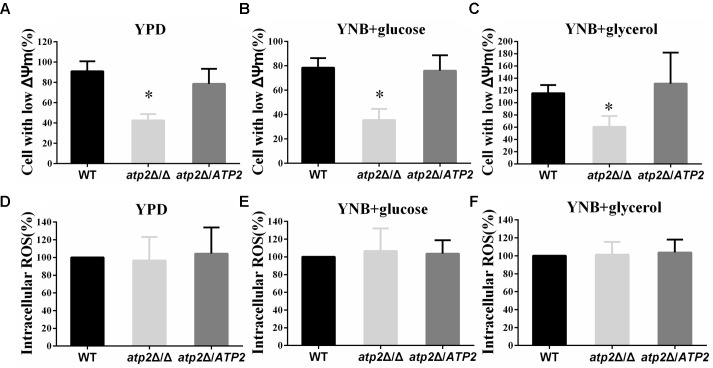
The mitochondrial membrane potential decreases and intracellular ROS is not altered in the *atp2*Δ/Δ. **(A–C)** Mitochondrial membrane potential was measured with JC-1 dye by flow cytometry. Cells were collected, resuspended in PBS and incubated with JC-1 at 37°C for 15 min. Gated region R1 includes cells with intact mitochondrial membranes and gated region R2 depicts cells with loss of mitochondrial membrane potential. ^∗^*P* < 0.05 compared with WT. **(D–F)** The ROS levels were measured with DCFDA dye by flow cytometry. Cells were collected, resuspended in PBS and incubated with DCFDA at 37°C for 20 min. Representative results from at least three experiments are shown.

Deletion of *ATP2* apparently impairs the function of mitochondrial function. Using the JC-1 fluorescent dye, we found higher numbers of non-polarized cells in *atp2*Δ/Δ mutant when grown overnight in YPD, YNB with glucose or YNB with glycerol media overnight (*p* < 0.05) when compared with WT and the *atp2*Δ*/ATP2* strain (**Figures 5A–C**). The number of non-polarized cells was similar between the *atp2*Δ*/ATP2* strain and WT. The hypopolarization of mitochondria was consistent with declined cellular ATP content of the *atp2*Δ/Δ mutant at the later stage described above. Since dysfunctional mitochondrion usually increases ROS, we wondered if deletion of *ATP2* also elevates the intracellular ROS in *C. albicans*. Our results showed that the ROS levels from the *atp2*Δ/Δ mutant grown in YPD, YNB-glucose and YNB-glycerol were similar to those in WT and *atp2*Δ*/ATP2* strains (**Figures 5D–F**). These results indicate that deletion of *ATP2* reduces the conversion of ADP to ATP but has limited impact on electron transfer.

### Deletion of *ATP2* Results in Avirulence in a Mouse Model of Hematogenously Disseminated Candidiasis

**FIGURE 6:**
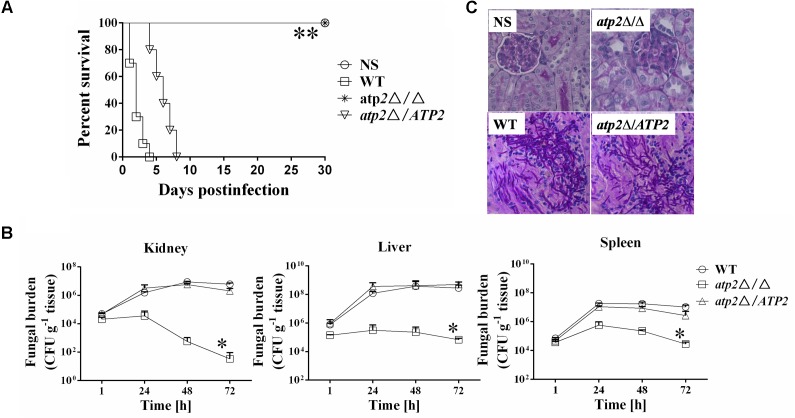
Deletion of *ATP2* results in avirulence in a mouse model of hematogenously disseminated candidiasis. **(A)** Mice survival following infection with 1 × 10^6^ cells of the *Candida albicans*. ^∗∗^*P <* 0.0001 compared to mice infected with WT. **(B)** Fungal burden (cfu/g tissue) of mice infected with 1 × 10^6^ cells of the *C. albicans*. Following infection, at 1, 24, 48, and 72 h, kidney, liver, and spleens were removed, weighed, homogenized and samples were plated on YPD agar medium. The total colony forming units (cfus) in tissue for each strain were shown over time. Data are from two experiments with three mice per time point for each strain (mean ± SD). ^∗^*P <* 0.05 compared to mice infected with WT. **(C)** Periodic acid Schiff staining of kidney sections from mice after 1 day of infection with *C. albicans*. Pictures were taken at 40 × magnification.

In a murine disseminated model, all of the mice (n = 10) infected intravenously with 1 × 10^6^ cells of *atp2*Δ/Δ survived 30 days post infection (**Figure 6A**). By contrast, mice infected with same number of WT cells began to die on day 1 post infection and all mice became moribund on day 4. At the same time, mice infected with 1 × 10^6^ cells of *atp2*Δ/*ATP2* began to die on day 4 and all mice were moribund on day 8. Clearly, *ATP2* is required for *C. albicans* virulence *in vivo*.

These virulence phenotypes of different strains are correlated well with their invasion in the host kidney, liver and spleen tissues (**Figure 6B**). At 1 h post infection, the colony forming units (cfus) were similar among three strains, which is then reduced by about one order of log-scale magnitude at 48 h post infection and further reduced by two orders of magnitude at 72 h post infection in mice that infected by the *atp2*Δ/Δ. By contrast, fungal cells gradually increased in kidneys of WT or *atp2*Δ/*ATP2* infected mice. The peaks of fungal load in liver and spleen were 48 h post infection for either WT or *atp2*Δ/*ATP2* infection and 24 h for *atp2*Δ/Δ infected mice. Compared to the control strains, the cfu numbers in both tissues were declined fast in the mutant strain from 24 to 72 h post infection. The significant reduction of fungal burden in the organs of mice infected with the *atp2*Δ/Δ within 3 days suggests that *ATP2* is required for *C. albicans* invasion and persistence in these vital organs.

The low fungal burden in the kidneys of mice infected with the *atp2*Δ/Δ was verified by histopathology as well. In the kidneys of mice infected with either WT or *atp2*Δ/*ATP2*, micro-abscesses consisting of numerous inflammatory cells were often surrounded by abundant hyphae or pseudohyphae (**Figure 6C**) at 24 h post infection, which is scarce in *atp2*Δ/Δ infected mice.

### Transcriptional Changes in the *atp2*Δ/Δ Mutant

**FIGURE 7:**
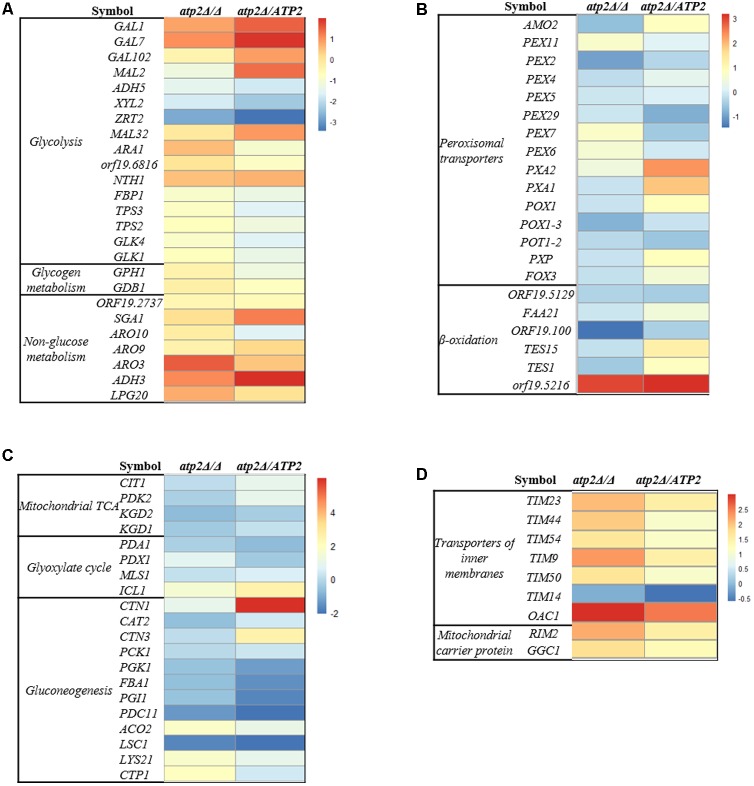
Transcriptional profiling of carbon metabolism and mitochondria in the *atp2*Δ/Δ. Up- and down-regulated genes in the *atp2*Δ/Δ mutant and *atp2*Δ*/ATP2* versus to WT are coded from white to black as indicated. Four functional gene groups are shown: **(A)** glycolysis, glycogen metabolism, non-glucose metabolism and the Ehrlich pathway of which amino acids are used as carbon sources; **(B)** mitochondrial TCA and glyoxylate cycle and gluconeogenesis; **(C)** peroxisomal transporters, β-oxidation; **(D)** mitochondria CI-CV complexes, transporters of inner membranes (TIMs) and transporters of outer membrane (TOMs) of mitochondria, mitochondrial ETC assembly and ion channels (magnesium, phosphate).

The transcriptional response of *C. albicans* was performed by Illumina sequencing and analysis of RNAseq data in *atp2*Δ/Δ mutant. When referred to the WT strain, a total of 45 genes were down-regulated, and 318 genes were up-regulated in *atp2*Δ/Δ mutant. We focused on genes that were related to carbon metabolism and mitochondrial functions, which were summarized in **Figures 7A–D**.

In regard to the carbon metabolism, hexose transporter (D-xylulose reductase, *XYL2*) and the iron transporter (*ZRT2*) were 3.34- and 6.77-fold down-regulated in the *atp2*Δ/Δ mutant, respectively (**Figure 7A**). In addition, an ARO gene (*ARO3*) was decreased significantly in *atp2*Δ/Δ mutant. Generally speaking, transcriptional levels of genes for alternative carbon pathways were minor or no changed. As for genes encoding for tricarboxylic acid (TCA) cycle, *LSC1* was significantly down-regulated (0.31) but other genes (*ACO2, LYS21, CTP1*) were significantly up-regulated from 3.14- to 3.73-fold in the mutant strain (**Figure 7B**). The up-regulated genes in the *atp2*Δ/Δ mutant also included genes for β-oxidation of fatty acids and peroxisomal transporters. For example, genes for the fatty acid metabolism (orf19.5216) and the glyoxylate cycle (*ICL1*) were up-regulated 7.89- and 2.81-fold, respectively (**Figures 7B,C**). It appeared that deletion of *ATP2* favors the TCA cycle, fatty acid metabolism and glyoxylate cycle, which was consistence with highly expressed mitochondrial functions. We found that the genes encoding for mitochondrial ETC proteins of CI, CIII, CIV and CV subunit proteins, ETC protein complex assembly, transporters of several TIM proteins (*TIM23, TIM44, TIM54, TIM9, TIM50, TIM14*) were up-regulated 3.12- to 6.15- fold. In addition, other mitochondrial transporters (*RIM2, GGC1, OAC1*) were also up-regulated 3.36- to 7.94- fold (**Figure 7D** and Supplementary Figure S7). The glycolysis (*GAL1, GAL7*, *GAL102, MAL32, NTH1*), non-glucose metabolism (*SGA1, ADH3*) and gluconeogenesis (*CTN1*) were up-regulated in the *atp2*Δ/*ATP2*. Consistent with a higher mitochondrial energetic activity, the mitochondrial rRNA biogenesis along with cytoplasmic rRNA machinery in *atp2*Δ/Δ mutant were both up-regulated. These data suggest that a likely universal regulatory network is governing the mitochondrial energetic events due to the difficulty in ADP conversion in this mutant (Supplementary Figure S8).

### NADH/NAD^+^ Ratio Increased in the *atp2*Δ/Δ Mutant

**FIGURE 8:**
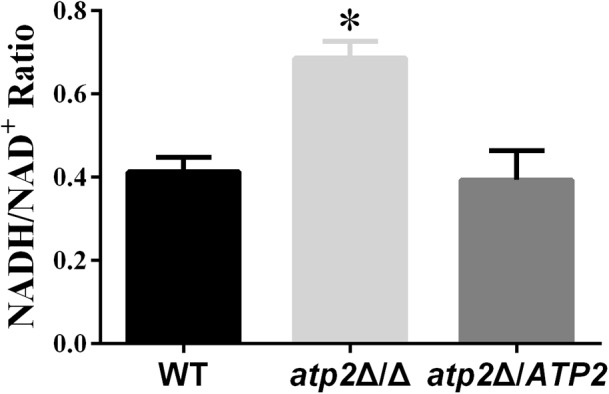
The redox balance in the *atp2*Δ/Δ. The strains were originally incubated in YPD medium and then shift to the same volume of YNB and continued to incubate at 30°C for 24 h. The NAD^+^ and NADH contents were measured. The results were the means of at least three independent experiments, and error bars represented the SDs. ^∗^*P <* 0.05 compared with WT.

In addition to changes occurring in the transcriptome, we also assessed the impact of *ATP2* on *C. albicans* redox balance. We determined the intracellular ratio of NADH, a central redox cofactor of the bacterial cell, versus its reduced version NAD^+^. NADH/NAD^+^ ratio increased in *atp2*Δ/Δ mutant (*p* < 0.05) when compared with WT and the *atp2*Δ*/ATP2* strain (**Figure 8**). This result indicates that deletion of *ATP2* induces the intracellular NADH/NAD^+^ redox balance to shift toward a reducing state.

## Discussion

In this study, the carbon metabolic and pathogenic roles of the *ATP2* gene in *C. albicans* are investigated *in vitro* by cell viability in different carbon sources and by virulence *in vivo*. The deletion of the *ATP2* gene leads to avirulence in murine candidiasis model and also leads to a delayed onset of reduced cell viability, the latter having been reported in *M. tuberculosis* when it was treated by F_1_F_o_-ATP synthase inhibitor (Andries et al., 2005; Koul et al., 2014). The growth and cell viability of *atp2*Δ/Δ mutant are normal during the first 5 days at least. The growth defects and decreased viability appear diverge after 5 days, which correlates to a same or higher cellular ATP content at first, and decreasing at later stages. We observed that a comparable initial ATP production in the *atp2*Δ/Δ mutant coupled with an even higher production over the first 3 days in YNB (when compared to YPD) suggests that the alternative energetic pathways must be highly tuned in YNB to produce adequate ATP for cell survival and growth. This highly tuned response of energetic pathway may also explain the extended survival. The declined cell viability after the reduced ATP production has been reported in *M. tuberculosis* when it was treated by F_1_F_o_-ATP synthase inhibitor (Koul et al., 2014). However, Even though the ATP production is an indicator of the number of viable microbial cells, our results are not conclusive concerning whether the cause of the decrease in cell viability is the decrease in ATP content or vice versa.

In the absence of glucose, the *atp2*Δ/Δ mutant displayed an immediately and more sharply declining cell viability in non-fermentable carbon sources. These carbon sources are supposedly metabolized by three main pathways: β-oxidation of fatty acids, the glyoxylate cycle and gluconeogenesis, which differ from glycolysis operated by a fermentable carbon source. Although the *atp2*Δ/Δ displayed a higher viability in glucose than in alternative carbon source, transcriptional data show no changes in the glycolysis pathway. This observation is also seen in *in vitro* studies of the reduced cell viability of *M. smegmatis* in glucose and glycerol supplemented with F_1_F_o_-ATP synthase inhibitor (Hards et al., 2015). Indeed, when *M. tuberculosis* is growing on non-fermentable energy sources, it is more rapidly killed by F_1_F_o_-ATP synthase inhibitor. We believe that utilization of the alternative carbon sources may contribute to maintaining a part of ATP production for *atp2*Δ/Δ mutant cell to survive, which is supported by the up-regulated TCA, β-oxidation and peroxisomal transporter in the transcriptome. However, these alternative energy pathways are apparently not sufficient when F_1_F_o_-ATP synthase is continually inhibited. Taken together, the ability of *C. albicans* to synthesize ATP by glycolysis can prevent rapid lethality in fermentable carbon sources. That is, without the ability to synthesize ATP either by glycolysis or by respiratory pathway, lethality in *C. albicans* would quickly follow.

The fungal loads of *atp2*Δ/Δ mutant *in vivo* increase over 24 h post infection, but drop sharply in kidney and gradually reduce in liver and spleen, which is a little similar to cell viability of the mutant *in vitro* on non-fermentable carbon sources. Recently, it has been reported that, except for liver and brain, *C. albicans* in other tissues uses alternative metabolic pathways to utilize host proteins, amino acids, lipids, and phospholipids (Mayer et al., 2013). Ramirez and Lorenz (2007), also reported that *C. albicans* often encounters carbon-poor conditions during growth in the host and that the ability to efficiently utilize multiple non-fermentable carbon sources is a virulence determinant. New data suggest that *C. albicans* acts differently from *S. cerevisiae* with regard to control of carbon metabolism (Sandai et al., 2012). The premise of these data is that non-fermentable carbon sources assimilation in *C. albicans* continues even after glucose is available, which allows *C. albicans* to adapt to a host niche where carbon availability may change over time. Mitochondria play an important role in *C. albicans* pathogenicity (Shingu-Vazquez and Traven, 2011; Calderone et al., 2015), and are intimately related to the assimilation of non-fermentable carbon sources. This in turn may explain the avirulence we see in the mutant.

The changes in the ΔΨm, energy metabolism and ROS production have been linked to cell fate (Martinez-Reyes and Cuezva, 2014). We detect a decrease of ΔΨm but no change in production of ROS in the *atp2*Δ/Δ mutant, which is different from increases in ΔΨm and ROS production mediated by F_1_F_o_-ATP synthase inhibition in human carcinomas (Formentini et al., 2012). Besides, the ΔΨm and the production of ROS are not correlated in *Mycobacterium* (Hards et al., 2015; Lamprecht et al., 2016) and *Salmonella* (Lee et al., 2013) by inhibiting F_1_F_o_-ATP synthase. The results of decrease in ΔΨm and unchanged ROS in *atp2*Δ/Δ mutant are apparently contradictory for mitochondrial function from other species. We reason that β subunit is a component of F_1_ complex of the F_1_F_o_-ATP synthase, which catalyzes the conversion of ADP to ATP but has limited impact on electron transfer. So the mitochondrial function of *atp2*Δ/Δ mutant is more consistent with an uncoupler-like mode of action, which leads an increase in futile proton conductance into the matrix but does not cause any back pressure to substantially impede ETC activity for electron leakage. In support of this idea, an increase in futile proton conductance, together with decreases in cellular ATP content and ΔΨm, and an unchanged ROS have recently been reported with classical uncouplers (Lee et al., 2013; Lamprecht et al., 2016).

**FIGURE 9:**
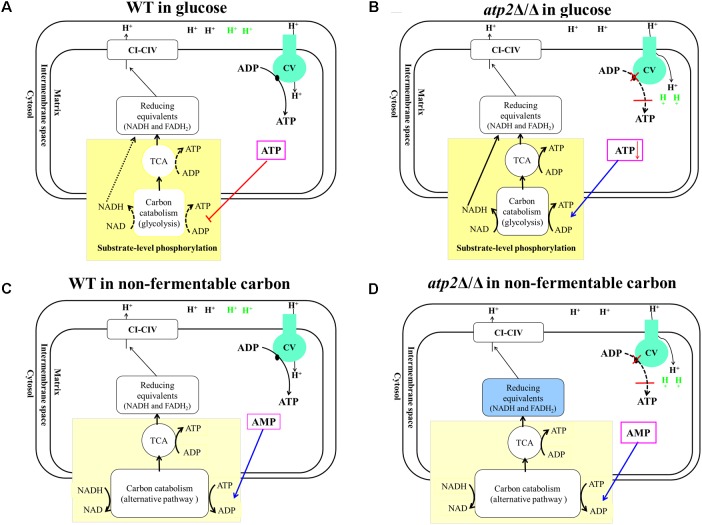
Proposed models of metabolic reaction after F_1_F_o_-ATP synthase β subunit inhibition. **(A)** In glucose, WT produces ATP via glycolysis and oxidative phosphorylation. ATP level mainly regulates both glycolysis and the TCA cycle via feedback inhibition to maintain energy homeostasis. **(B)** In the presence of glucose, *atp2*Δ/Δ mutant causes an uncoupler-like mode of action which leads an increase in futile proton conductance into the matrix, resulting in decreasing ATP synthesis. The declined ATP levels relieve carbon catabolism and feedback inhibition of the TCA cycle. As the feedback loop is interrupted by deletion of β subunit, this control system fails to regulate activity appropriately that stimulates glycolysis and the TCA cycle to their maximum paces. **(C)** In non-fermentable carbon sources, the carbon catabolism was regulated via AMP, and WT produces ATP only via oxidative phosphorylation. **(D)** In non-fermentable carbon sources, the *atp2*Δ/Δ mutant causes an uncoupler-like mode of action that disrupts both substrate level phosphorylation and oxidative phosphorylation that will abolish ATP production.

Normally, both glycolysis and the TCA cycle are mainly regulated by high intracellular ATP levels via feedback inhibition of energy-generating pathways in an effort to maintain energy homeostasis (**Figure 9A**). The action of this classic negative feedback loop normally serves to increase ATP production via both substrate level phosphorylation (carbon catabolism and TCA) and oxidative phosphorylation, thus returning the cell to energy balance. This has been characterized in *S. cerevisiae* (Larsson et al., 2000) and other organisms (Sanwal, 1970; Kemp and Foe, 1983; Jetten et al., 1994; Koebmann et al., 2002). According to this model, ATP reduction by lack of F_1_F_o_-ATP synthase β subunit would relieve feedback inhibition of energy-generating pathways, which results in stimulating the carbon catabolism and TCA to produce much more ATP. The stimulating carbon catabolism and TCA would increase reducing equivalents, which has been demonstrated by the result of increased NADH/NAD^+^ ratio in *atp2*Δ/Δ mutant and the studies, in which inhibition of F_1_F_o_-ATP synthase resulted in an increased NADH/NAD^+^ ratio in *M. tuberculosis* (Koul et al., 2014; Lamprecht et al., 2016). The sustainable increase in production of reducing equivalents eventually effects H^+^ dehydrogenized from metabolic substances, resulting in no more ATP production via substrate level phosphorylation (**Figure 9B**). However, this feedback system is not expected to be effective during growth on non-fermentable carbon sources. Instead, the carbon catabolism is regulated mainly by high intracellular AMP levels via activation of alternative energy-generating pathways in an effort to maintain energy homeostasis (**Figure 9C**) (Prevost and Moses, 1967; Hsie et al., 1969; Sanwal, 1970). The *atp2*Δ/Δ mutant displays immediate onset of reduced ATP levels in non-fermentable carbon sources. The simplest explanation for this is that *C. albicans* produces ATP only via oxidative phosphorylation in non-fermentable carbon sources. When the function of F_1_F_o_-ATP synthase is blocked in the *atp2*Δ/Δ mutant, dysfunctional oxidative phosphorylation will abolish ATP production, thereby leading to an effective decline in cell viability (**Figure 9D**).

## Conclusion

Inhibition of F_1_F_o_-ATP synthase β subunit could be responsible for the failure of *C. albicans* infection by disrupting carbon flexibility, which highlights the importance of carbon metabolism inhibitors in combating *C. albicans* in lipid- and amino acid-rich micro-environments. As such, the F_1_F_o_-ATP synthase may then be a target for therapeutic intervention.

## Author Contributions

S-XL, K-JZ, D-ML, and HZ designed the research plan. S-XL, H-TW, Y-SZ, Y-YJ, and Y-TL executed the experiments. S-XL, H-TW, Y-SZ, Y-YJ, Y-TL, K-JZ, D-ML, W-DL, and HZ performed the data analyses and writing. All authors read and approved the final manuscript.

## Conflict of Interest Statement

The authors declare that the research was conducted in the absence of any commercial or financial relationships that could be construed as a potential conflict of interest.
