# Supplementary material for: The F1Fo-ATP Synthase β Subunit Is Required for Candida albicans Pathogenicity Due to Its Role in Carbon Flexibility
Source: Front Microbiol. 2018 May 23;9:1025. doi: 10.3389/fmicb.2018.01025 (PMC5974098; doi:10.3389/fmicb.2018.01025)
Supplement: Supplementary file 1 [file Data_Sheet_1.doc]

Table S1. Strains used in this study.

| Strain | Parent | Genotype | Reference |
| --- | --- | --- | --- |
| SC5314 |  | Wild-type strain | [Gillum *et al*., 1984] |
| *atp2*Δ/Δ | SC5314 | [orf1](http://www.candidagenome.org/cgi-bin/locus.pl?locus=orf19.6854&seq_source=C. albicans SC5314 Assembly 21)[9.5653](http://www.candidagenome.org/cgi-bin/locus.pl?locus=orf19.5653&seq_source=C. albicans SC5314 Assembly 21)-1Δ::FRT/[orf1](http://www.candidagenome.org/cgi-bin/locus.pl?locus=orf19.6854&seq_source=C. albicans SC5314 Assembly 21)[9.5653](http://www.candidagenome.org/cgi-bin/locus.pl?locus=orf19.5653&seq_source=C. albicans SC5314 Assembly 21)-2Δ::FRT | This study |
| *atp2*Δ/*ATP2* | *atp2*Δ/Δ | [orf1](http://www.candidagenome.org/cgi-bin/locus.pl?locus=orf19.6854&seq_source=C. albicans SC5314 Assembly 21)[9.5653](http://www.candidagenome.org/cgi-bin/locus.pl?locus=orf19.5653&seq_source=C. albicans SC5314 Assembly 21)-1Δ::FRT/[orf1](http://www.candidagenome.org/cgi-bin/locus.pl?locus=orf19.6854&seq_source=C. albicans SC5314 Assembly 21)[9.5653](http://www.candidagenome.org/cgi-bin/locus.pl?locus=orf19.5653&seq_source=C. albicans SC5314 Assembly 21)-2::FRT | This study |

*Table S2. Primers used in this study for the deletion of ATP2 in C. albicans.*

| Primers |  | Sequence ^a^ |  |
| --- | --- | --- | --- |
| ATP2-1 |  | 5’-CCCgggcccTCAGAATGCGTTTGCCCTAT-3’ |  |
| ATP2-2 |  | 5’-CCCctcgagGAATGCAGCACGTGAACTGG-3’ |  |
| ATP2-3 |  | 5’-TCCccgcggTATCCCAGGTAGATTAGT-3’ |  |
| ATP2-4 |  | 5’-GCGgagctcAAGATCTAAATAAGTCTCG-3’ |  |
| CassetF |  | 5’-GCTTTCGGTCGCTGTTCTCA-3’ |  |
| CassetR |  | 5’-TGTTAGGCGTCATCCTGTGC-3’ |  |
| ATP2-5  ATP2-6  ATP2-7  ATP2-8  ATP2-F  ATP2-R  ATP2-11  ATP2-12 |  | 5’-GAGGAGGCTAAGAAGGTTGC -3’  5’-CAATGAAATCCAGACAGTCG -3’  5’-GCACCGAGGCAAAGGAGT -3’  5’-GTCCCAGCCAATGTCCAG -3’  5’- TTTCTGTGCGAGGGACTT-3’  5’- TAACCGACAGCAGATGGA -3’  5’- CCAAATAAGAATGAGGAGGCT -3’  5’- TCCGGCAATAACTGGCGATC -3’ |  |

^a^ Underlined sequences represent the following restriction sites: ApaI for ATP2-1, XhoI for ATP2-2, SacII for ATP2-3, SacI for ATP2-4.

The confirmation of the *atp2*Δ/Δ mutant and reconstituted strain (*atp2*Δ/*ATP2)* are performed via nested PCR (we made a diagram to show the primer sets location) and Southern blot as shown in **Figure S1, S2, S3, S4, S5 and S6**.

First, we used primer set which detected the correct cassette integration in *Candida albicans* cell (Figure S2). The PCR results showed that amplified stripe at 1404 bp with primer CassetF+CassetR (Figure S3A) represented that cassette integration was successfully transfected into *Candida albicans* cell; amplified stripe at 768 bp with primer ATP2-5+ATP2-6 (Figure S3B) represented that cassette integration was correctly transfected into 5’ fragments of *Candida albicans* cell; amplified stripe at 937 bp with primer ATP2-7+ATP2-8 (Figure S3C) represented that cassette integration was correctly transfected into 3’ fragments of *Candida albicans* cell. These results indicate that the cassette integration was correctly transfected into target gene *ATP2* locus of *Candida albicans*.

Second, we used a primer set to identify the disruption of target gene *ATP2* (Figure S4). As Figure S5A showed, amplified with primer ATP2-F+ATP2-R, E is without stripe for *atp2*Δ/Δ, F and f are stripes at 891 bp for WT and *atp2*Δ/*ATP2,* respectively. Amplified with primer P11+P12, G is stripe at 1633 bp for *atp2*Δ/Δ, H and h are stripe at 3558 bp for WT and *atp2*Δ/*ATP2,* respectively. These results suggest the *ATP2* null mutant and reconstituted strain are constructed successfully.

Third, we used primer set which detected the cassette integration discarded from *Candida albicans* cell (Figure S4). As Figure S5B showed, amplified no stripes with primer CassetF+CassetR, represented that cassette integration was discarded from *Candida albicans* cell (a-f).

In another way, Figure S6A is the result of Southern analysis of HindIII-digested genomic DNA of the parent strain WT, *atp2*Δ/Δ and *atp2*Δ/*ATP2* with the *ATP2*-specific probe. The result of Southern blot showed that *atp2*Δ/Δ was without hybridizing fragment, WT and *atp2*Δ/*ATP2* were hybridizing fragments in 38 kb*,* respectively. These results suggest the *ATP2* null mutant and reconstituted strain are constructed successfully. Figure S6B is the result of Southern analysis of SacI-digested genomic DNA of the parent strain WT, *atp2*Δ/Δ and *atp2*Δ/*ATP2* with the cassette integration-specific probe. The plasmid pSFS2 was hybridizing fragments in 543 bp; But WT, *atp2*Δ/Δ and *atp2*Δ/*ATP2* are without hybridizing fragment, respectively. The results represent that cassette integration was discarded from *Candida albicans* cell.


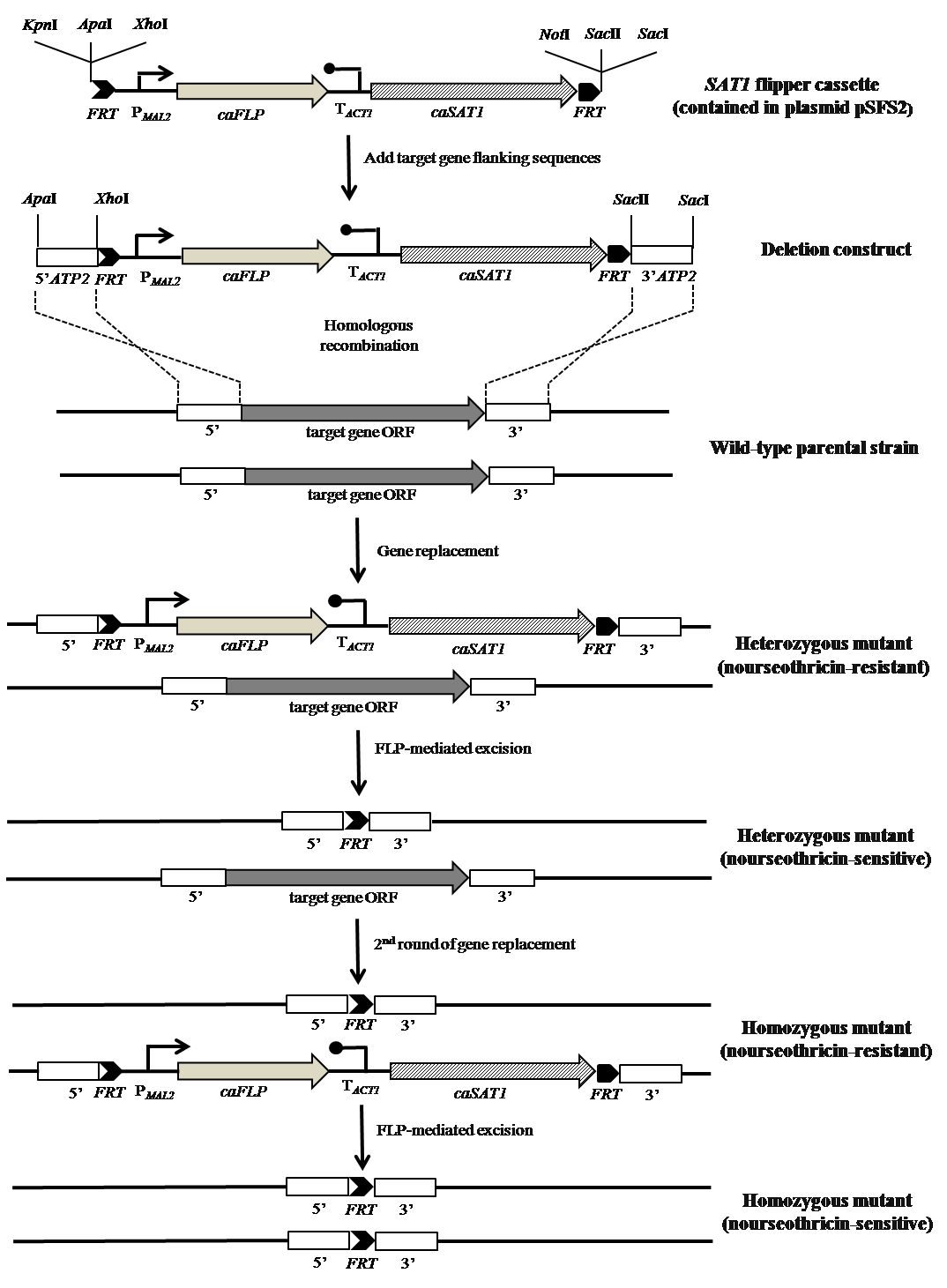


Figure S1. Schematic diagram of gene disruption by the SAT1-Flipper method.


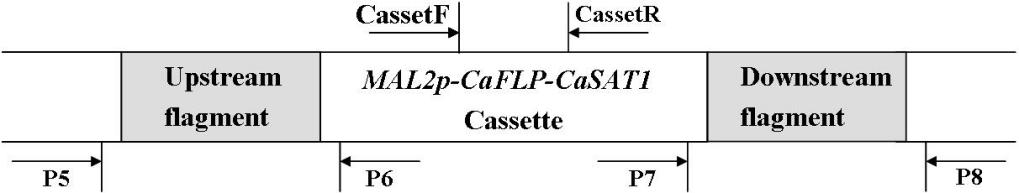


Figure **S2**. **Schematic diagram of the primers used to identify the correct cassette integration in *Candida albicans*.**


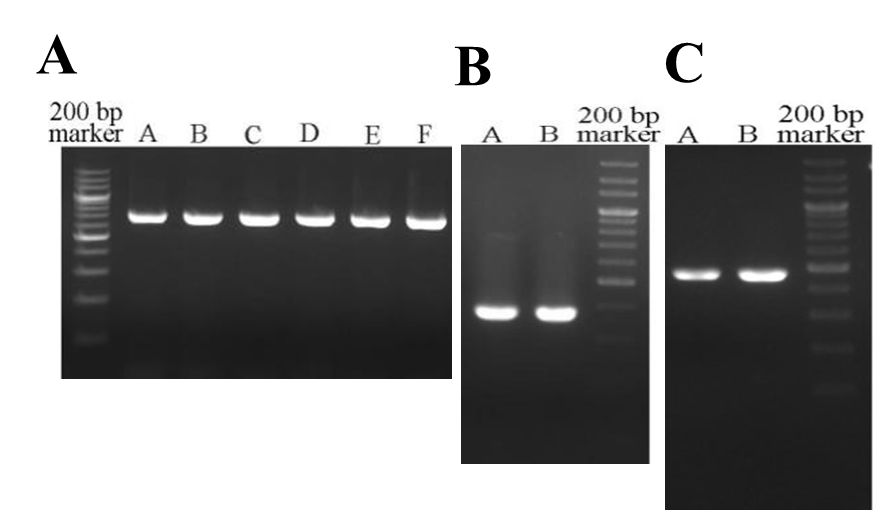


Figure S3. Identification of the cassette integration correctly transfected into *Candida albicans* cell. (A) Marker fragments of the *ATP2* knockout strains. (B) Upstream fragments of the *ATP2* knockout strains. (C) Downstream fragments of the *ATP2* knockout strains.


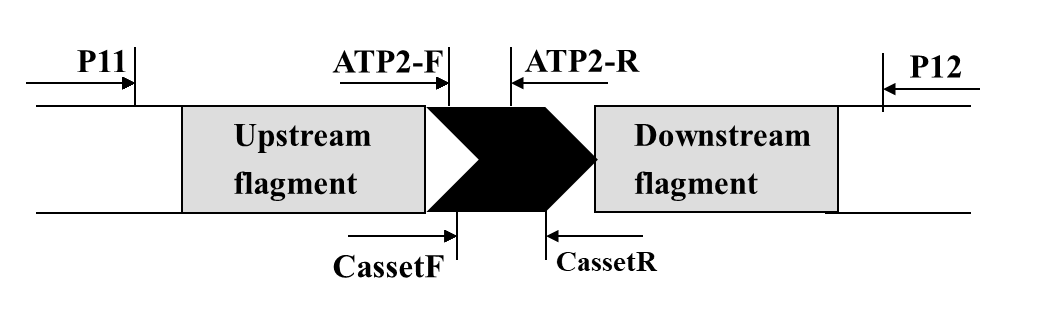


Figure S4. Schematic diagram of the primers used to identify the disruption of target gene *ATP2*.


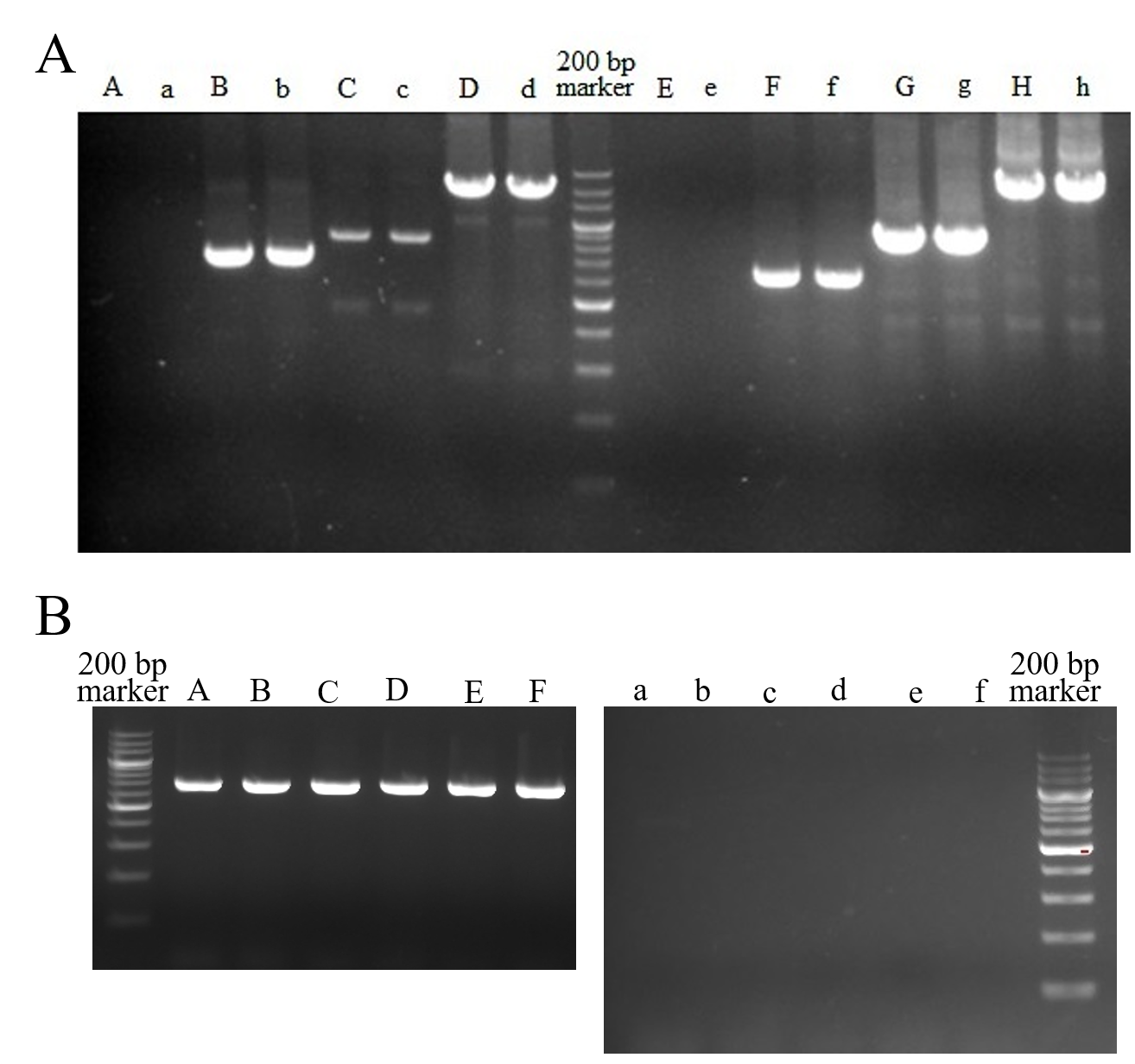


Figure S5. (A) Identification of the *ATP2* knockout and reconstituted strains. Primers used in this work were *ATP2* inside Check and outside Check. Amplified with primer ATP2-F+ATP2-R, E is without stripe for *atp2*Δ/Δ, F and f are stripes at 891 bp for WT and *atp2*Δ/*ATP2,* respectively. Amplified with primer P11+P12, G is stripe at 1633 bp for *atp2*Δ/Δ, H and h are stripe at 3558 bp for WT and *atp2*Δ/*ATP2,* respectively. These results suggest the *ATP2* null mutant and reconstituted strain are constructed successfully. (B) Identification of cassette integration. Amplified stripe at 1404 bp represented that cassette integration was successfully transfected into *Candida albicans* cell (A-F), and no stripe represented that cassette integration was discarded (a-f).


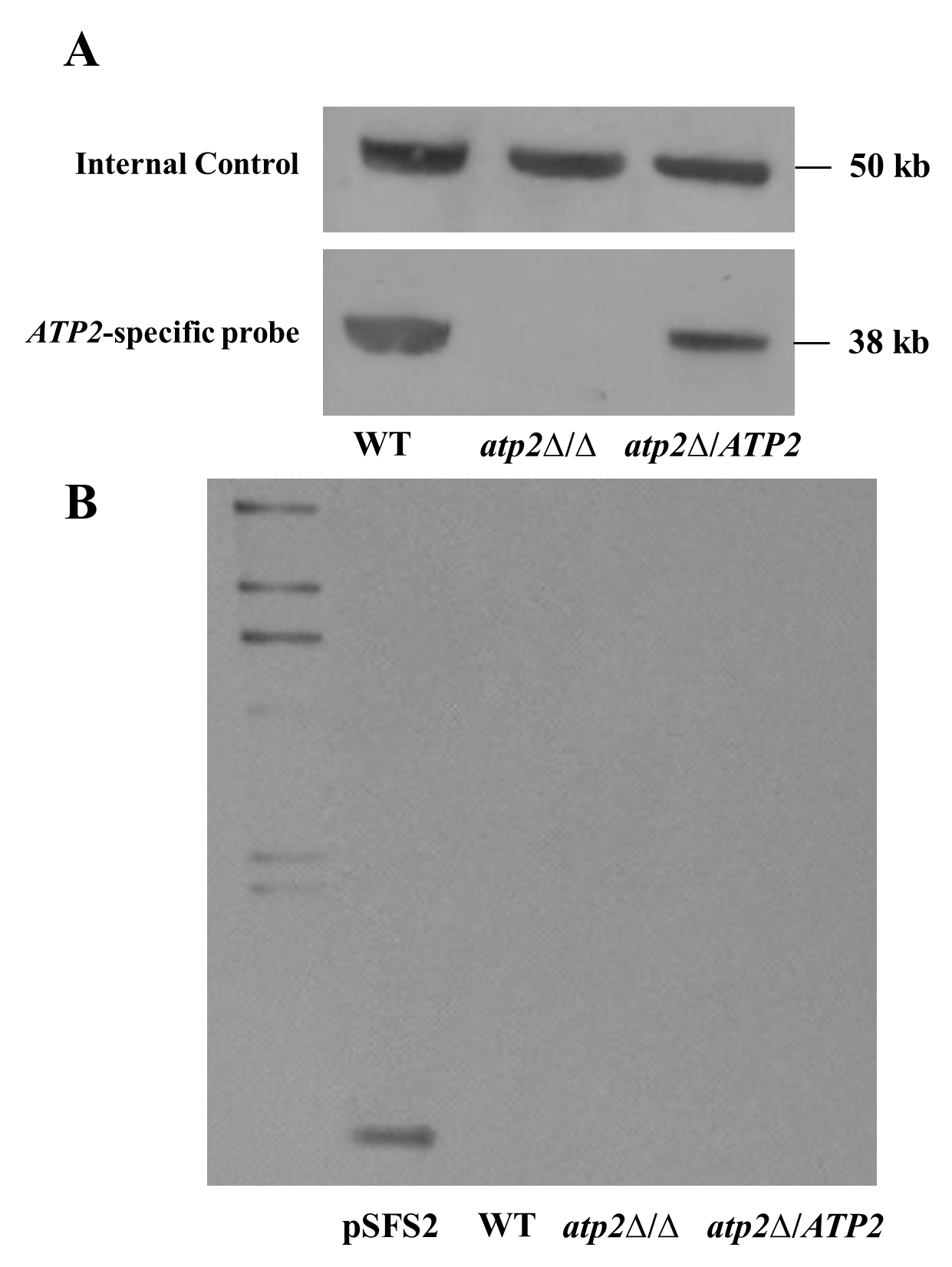


Figure S6. (A) Southern analysis of HindIII-digested genomic DNA of the parent strain WT, *atp2*Δ/Δ and *atp2*Δ/*ATP2* with the *ATP2*-specific probe. WT was hybridizing fragment in 38 kb; *atp2*Δ/Δ was without hybridizing fragment; *atp2*Δ/*ATP2* was hybridizing fragment in 38 kb. (B) Southern analysis of SacI -digested genomic DNA of the parent strain WT, *atp2*Δ/Δ and *atp2*Δ/*ATP2* with the cassette integration-specific probe. The plasmid pSFS2 was hybridizing fragments in 543 bp; But WT, *atp2*Δ/Δ and *atp2*Δ/*ATP2* are without hybridizing fragment, respectively.


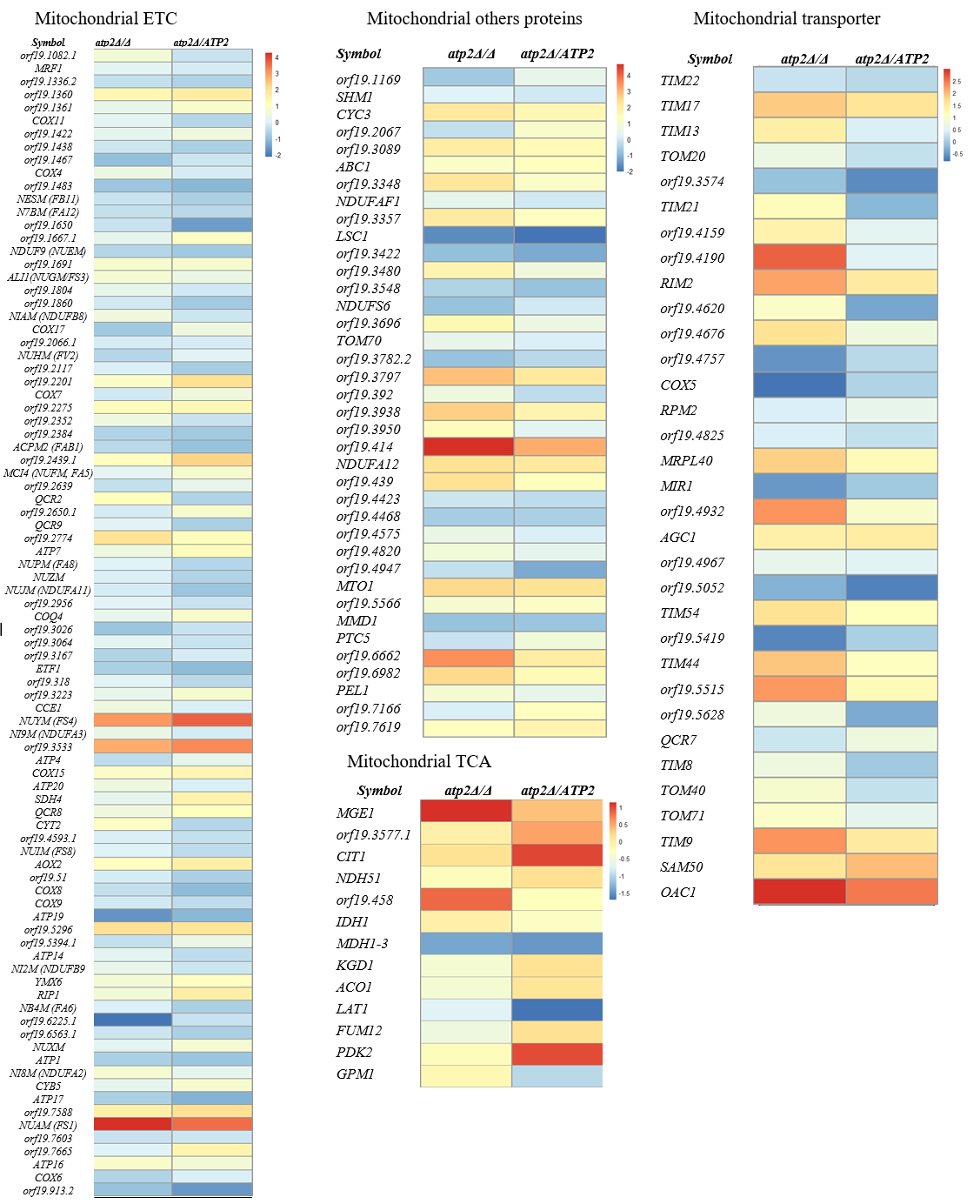


Figure S7. Transcriptional profiling of mitochondria CI-CV complexes, transporters of inner membranes (TIMs) and transporters of outer membrane (TOMs) of mitochondria, mitochondrial ETC assembly and ion channels (magnesium, phosphate) in the *atp2*Δ/Δ.


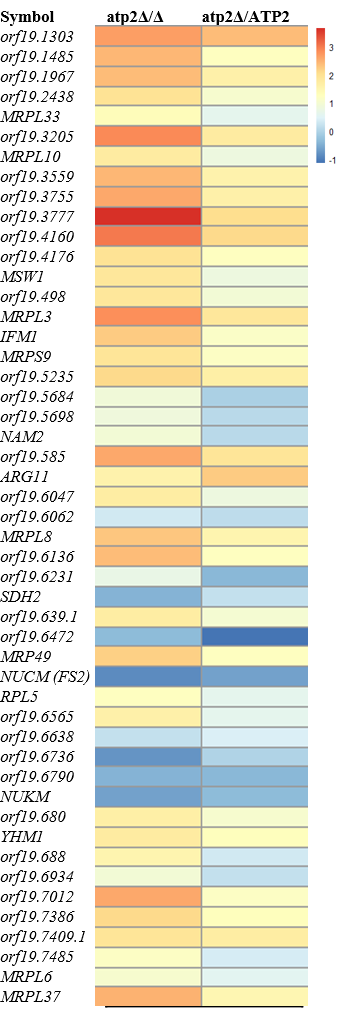


Figure S8. Transcriptional profiling of mitochondrial rRNA biogenesis in the *atp2*Δ/Δ.
